# EEG-ChTABNet: A Dual-Branch Channel-Wise Transformer with Gated Attention-Branch Network for EEG-Based Classification of Dementia

**DOI:** 10.3390/biomedicines14061345

**Published:** 2026-06-15

**Authors:** Noor Kamal Al-Qazzaz, Sawal Hamid Bin Mohd Ali, Siti Anom Ahmad

**Affiliations:** 1 Department of Biomedical Engineering, Al-Khwarizmi College of Engineering, University of Baghdad, Baghdad 47146, Iraq; 2 Department of Electrical, Electronic and Systems Engineering, Faculty of Engineering and Built Environment, Universiti Kebangsaan Malaysia, Bangi 43600, Selangor, Malaysia; sawal@ukm.edu.my; 3 Centre of Advanced Electronic and Communication Engineering, Department of Electrical, Electronic and Systems Engineering, Universiti Kebangsaan Malaysia, Bangi 43600, Selangor, Malaysia; 4 Department of Electrical and Electronic Engineering, Faculty of Engineering, Universiti Putra Malaysia, Serdang 43400, Selangor, Malaysia; sanom@upm.edu.my; 5 Malaysian Research Institute of Ageing (MyAgeing) TM, University Putra Malaysia, Serdang 43400, Selangor, Malaysia

**Keywords:** EEG, dementia, entropy, transformer, channel attention, deep learning, classification

## Abstract

**Background/Objectives**: Early and accurate discrimination of neurological conditions, dementia, stroke and healthy aging, remains a critical clinical challenge. Electroencephalography (EEG) is a non-invasive measure of brain dynamics and entropy-based features obtained from multichannel EEG have shown strong discriminative ability. However, existing deep learning approaches do not sufficiently address the combined challenges of small clinical cohorts and high-dimensional entropy feature spaces. In this study, a novel architecture is proposed for multi-class neurological EEG classification under extreme small-sample conditions. **Methods**: A novel dual-branch Channel-wise Transformer and Attention-Branch Network (EEG-ChTABNet) are pr to classify 19-channel EEG entropy features into three classes (dementia, stroke, healthy control; N = 45; 15 per class). The architecture suggests four new designs. First, the Channel Importance Attention (CIA) block, which adaptively learns to re-weight the importance of electrodes via squeeze-excitation. Second, the dual-branch encoder, which combines the global multi-head self-attention with the local depthwise-separable convolution. Third, the gated sigmoid fusion mechanism. Fourth, the bottleneck residual classification head, to solve overfitting. Eight entropy feature sets: Amplitude-Aware Permutation Entropy (AAPE), Attention Entropy (AttEn), Dispersion Entropy (DisEn), Distribution Entropy (DistrEn), Fluctuation-based Dispersion Entropy (FDispEn), Fuzzy Entropy (FuzEn), Linear Gaussian Estimation of the Conditional Entropy (LinEn), and Symbolic Dynamics (SyDy) were evaluated individually with stratified 5-fold cross-validation on within-fold SMOTE augmentation. **Results**: EEG-ChTABNet consistently outperformed the baseline Transformer on all 8 feature sets. DisEn and SyDy features yielded peak classification accuracy of 73.3% (AUC: 0.823 and 0.857, respectively) compared to the corresponding baseline of 57.8% and 55.6%. SyDy achieved the best overall AUC of 0.857 and the dementia detection sensitivity was up to 86.7% over multiple feature sets. **Conclusions**: EEG-ChTABNet shows the effectiveness of channel-adaptive, dual-branch Transformer Designs for EEG-based neurological classification from Small-Sample Entropy Feature Data, and Identifying SyDy and DisEn as the Most Discriminative Feature Representations for Three-Class Neurological EEG Classification.

## 1. Introduction

Neurological disorders represent a growing health burden in the world, including Alzheimer’s disease (AD) and other related dementias, as well as cerebral vascular diseases such as stroke. The World Health Organization estimates that dementia is a problem affecting about 55 million individuals all over the world, with stroke being the second cause of death and one of the major causes of long-term disability [1]. The correct and prompt distinction of these conditions as compared to each other and to other healthy aging processes is of utmost clinical significance, but it presents a significant diagnostic problem in virtue of the similarity of symptoms and the constraints of standardized clinical tests [2,3].

One neuro-recording modality that has proven especially viable to this end has been electroencephalography (EEG). It has a number of practical benefits over other imaging methods: it is non-invasive, and it is widely available, inexpensive, and can record the rapid dynamics of neural activity at millisecond time resolution [2,4]. More importantly, EEG signals become indicators of changes in the complexity and regularity of the degradation of the neurodegenerative process and ischemic injury.

Entropy-based and nonlinear complexity measures have been defined as effective descriptors of these changes, and they are indications of changes in the underlying mechanisms of neural information processing [5]. Many measures of EEG complexity have been used as neurological classifiers, such as Sample Entropy, Approximate Entropy, Permutation Entropy, Dispersion Entropy, Fuzzy Entropy, and Symbolic Dynamics, to mention a few [6,7,8]. These measures are a measure of the various aspects of signal abnormalities and intricacy, particularly those calculated on the whole standard EEG channels and averaged across subjects; however, they represent compact but very informative tabular feature vectors, one scalar per channel, rather than time-series data [9].

Recent developments in attention-based models, especially Transformer models, have shown impressive performance in learning highly involved dependencies in sequential and tabular information [10]. A number of Transformer variants that have been appropriated to analyze EEG have been introduced, such as the Bilinear Attention Feature Temporal Convolutional Network (BAFTCNet) [11] and the Deep Brain Transformer Spatial-Projection Net (DBTSPNet) [12]. These architectures consider the channels of EEG as tokens and use self-attention to model inter-channel interactions. Their use in tabular entropy features, however, and their performance in the case of severe data scarcity are not well studied.

This study fills in these gaps with a number of important contributions:

1. The Channel-wise Transformer and Attention-Branch Network (EEG-ChTABNet), an innovative dual-branch Transformer architecture featuring a Channel Importance Attention (CIA) block, a gated fusion mechanism, and a bottleneck residual classification head, meticulously engineered, was presented for small-sample EEG entropy feature classification.
2. A systematic comparison of the proposed model with a typical Transformer baseline (BAFTCNet/DBTSPNet-inspired) using eight different sets of EEG entropy and complexity features was performed.
3. A strict assessment framework that includes stratified 5-fold cross-validation with within-fold SMOTE augmentation and a full set of measures, such as accuracy, sensitivity, specificity, precision, and AUC, was offered.
4. We illustrate which entropy feature set provides the greatest distinguishing representation for the three-class neurological classification task.

### Related Works

The work predominantly utilized handcrafted spectral characteristics extracted from conventional frequency bands (delta, theta, alpha, beta, and gamma), integrated with classical machine learning classifiers, including Support Vector Machines (SVMs) and Linear Discriminant Analysis (LDA) [13]. A slowing of the EEG, marked by higher theta/delta power and lower alpha power, has been repeatedly linked to Alzheimer’s disease and similar dementias [14]. Changes in EEG caused by a stroke include localized slowing, less activity at high frequencies, and uneven power distributions [15].

More recent research has focused on entropy and nonlinear complexity measurements, which capture features of the EEG that spectrum analysis overlooks. Permutation Entropy (PE) and its amplitude-aware variant (AAPE) have been utilized in the identification of Alzheimer’s disease, consistently indicating diminished complexity in affected individuals [6]. Fuzzy Entropy (FuzEn) and Sample Entropy (SampEn) have been employed to differentiate moderate cognitive impairment from normal aging [8]. Dispersion Entropy (DisEn), a newly developed metric, has exhibited notable sensitivity to alterations in EEG signal structure within neurological frameworks [16]. Symbolic patterns (SyDy) analysis has demonstrated efficacy in delineating the macrostructure of EEG patterns in both epileptic and neurodegenerative disorders [17].

The use of deep learning on EEG has expanded a lot, and convolutional neural networks (CNNs), recurrent networks (RNNs/LSTMs), and Transformers are all being used [10,18]. For raw EEG or spectro-temporal representations, CNNs like EEGNet [19] have set high standards. The DBTSPNet architecture [12] uses a dual-stream technique that combines temporal and spatial processing to classify motor images from EEG signals. BAFTCNet [11] included bilinear attention feature fusion into a temporal convolutional framework, showcasing enhanced discriminability on limited EEG datasets.

In this study, the transformer was designed on EEG to be able to capture long-range dependencies through self-attention, which makes it good for modeling relationships between electrodes in which each electrode is represented as a token, and attention weights can learn how important and related physically distributed recording sites are to each other [20]. Standard Transformers, on the other hand, have other problems when they are used on tabular feature data instead of raw signal sequences in which they do not have a meaningful positional ordering and need significant regularization when there are only a few samples [21].

A constant problem in clinical EEG investigations is that there are not enough well-labeled data. Clinical cohorts for rare or specialized illnesses typically consist of less than 100 cases, resulting in a small-N, moderate-to-high feature dimensionality context in which conventional deep learning methodologies exhibit significant overfitting [22]. Transfer learning, data augmentation, and ensemble approaches are some of the strategies that have been suggested to deal with this issue. Synthetic Minority Oversampling Technique (SMOTE) is a popular way to add more data to tables. It does this by creating synthetic samples by interpolating between existing samples in feature space. When used in cross-validation folds, SMOTE has been found to make classifiers work better on small clinical datasets [23]. Label smoothing, cosine learning rate scheduling, and early stopping are all regularization techniques that work well together and have been proven to make Transformer training more stable when data are limited [24].

## 2. Materials and Methods

**Figure 1:**
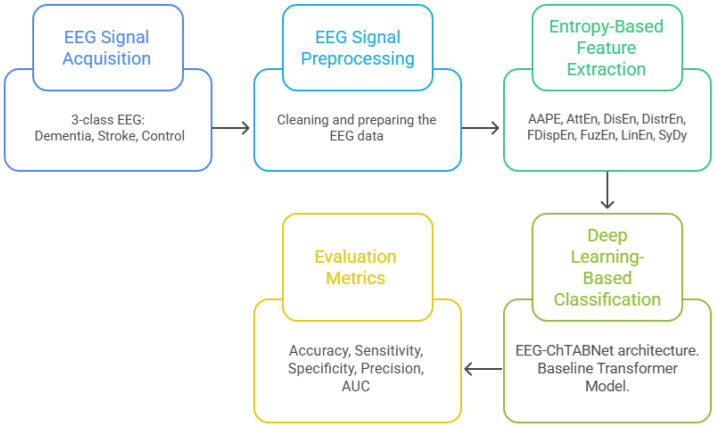
The pipeline of the proposed method.

The proposed framework for automated EEG-based neurological classification follows a structured multi-stage pipeline, as illustrated in Figure 1. The pipeline operates sequentially across four principal stages: firstly, EEG signal acquisition; secondly, preprocessing; thirdly, entropy-based feature extraction; Fourthly, deep learning-based classification using the proposed EEG-ChTABNet architecture.

### 2.1. EEG Signal Acquisition and Preprocessing

EEG data were collected from 45 individuals (15 with dementia, 15 with stroke, and 15 healthy controls) utilizing a conventional 19-channel cap that adheres to the worldwide 10–20 electrode placement method. EEG activity was recorded using the Nicolet One (V32) system during an auditory working memory task [25]. Accordingly, Pusat Perubatan Universiti Kebangsaan Malaysia enrolled participants in accordance with study protocols approved by the PPUKM Human Ethics Committee, and all participants signed an informed consent form.

A multi-step preprocessing pipeline was used to make sure that the signal was accurate and that any physiological or environmental artifacts were removed before feature extraction.

To keep therapeutically important brain oscillatory activity in the 0.5 to 64 Hz frequency range, a bandpass finite impulse response (FIR) filter was used first. This filter efficiently blocked DC drift, moderate baseline variations, and high-frequency noise, such as muscular artifacts. Notch filtering at 50 Hz was then used to get rid of interference from power lines.

After that, Independent Component Analysis with wavelet transform (ICA-WT) was used to find and get rid of eye and heart artifacts and denoise these artifacts. The continuous EEG recordings were split into non-overlapping epochs of 5 s of fixed length using an epoch-based windowing method. Each channel was processed separately, and the feature extraction stage took the preprocessed signals from all 19 channels as input.

### 2.2. Entropy-Based Feature Extraction

Eight time-domain entropy and nonlinear complexity measures were independently extracted from each of the 19 EEG channels for every subject, yielding eight separate 19-dimensional feature vectors per subject. These measures were selected to capture complementary facets of EEG signal regularity, distributional structure, and temporal dynamics that are known to be altered in neurological conditions. The eight features are as follows:

1. Amplitude-Aware Permutation Entropy (AAPE): An extension of standard permutation entropy that incorporates amplitude information into the ordinal pattern analysis, providing sensitivity to both the rank ordering and the magnitude of EEG fluctuations [26].
2. Attention Entropy (AttEn): A measure of the entropy of attention-weighted signal distributions, capturing the concentration or dispersion of signal energy across temporal patterns [27].
3. Dispersion Entropy (DisEn): Quantifies the diversity of amplitude dispersion patterns by mapping the signal into a set of symbolic classes and computing the entropy of the resulting pattern distribution [8,28,29].
4. Distribution Entropy (DistrEn): Measures the entropy of the empirical probability distribution of pairwise sample distances, providing a distributional characterization of signal complexity [30].
5. Fluctuation-based Dispersion Entropy (FDispEn): A refined variant of DisEn that incorporates local fluctuation information, improving sensitivity to transient and non-stationary signal components [8].
6. Fuzzy Entropy (FuzEn): Employs fuzzy membership functions to assess the regularity of template matching within the EEG signal, offering improved statistical stability relative to sample entropy [31,32].
7. Linear Gaussian Estimation of the Conditional Entropy (LinEn): Computes the entropy of the logarithmically transformed energy distribution of the EEG signal, capturing spectral energy spreading across temporal windows [33].
8. Symbolic Dynamics (SyDy): Encodes the EEG time series into a symbolic alphabet and analyzes the statistical structure of the resulting symbol sequences, reflecting the macrostructural temporal patterning of neural dynamics [27,34,35].

Each feature set was evaluated independently throughout the experimental pipeline, enabling a systematic comparison of the discriminative power of each entropy measure for the three-class neurological classification task.

### 2.3. Deep Learning-Based Classification

In this work, a novel dual-branch Channel-wise Transformer and Attention-Branch Network (EEG-ChTABNet) was presented for classifying EEG entropy features into three classes: dementia, stroke, and healthy control.

#### 2.3.1. Proposed Model: EEG-ChTABNet

**Figure 2:**
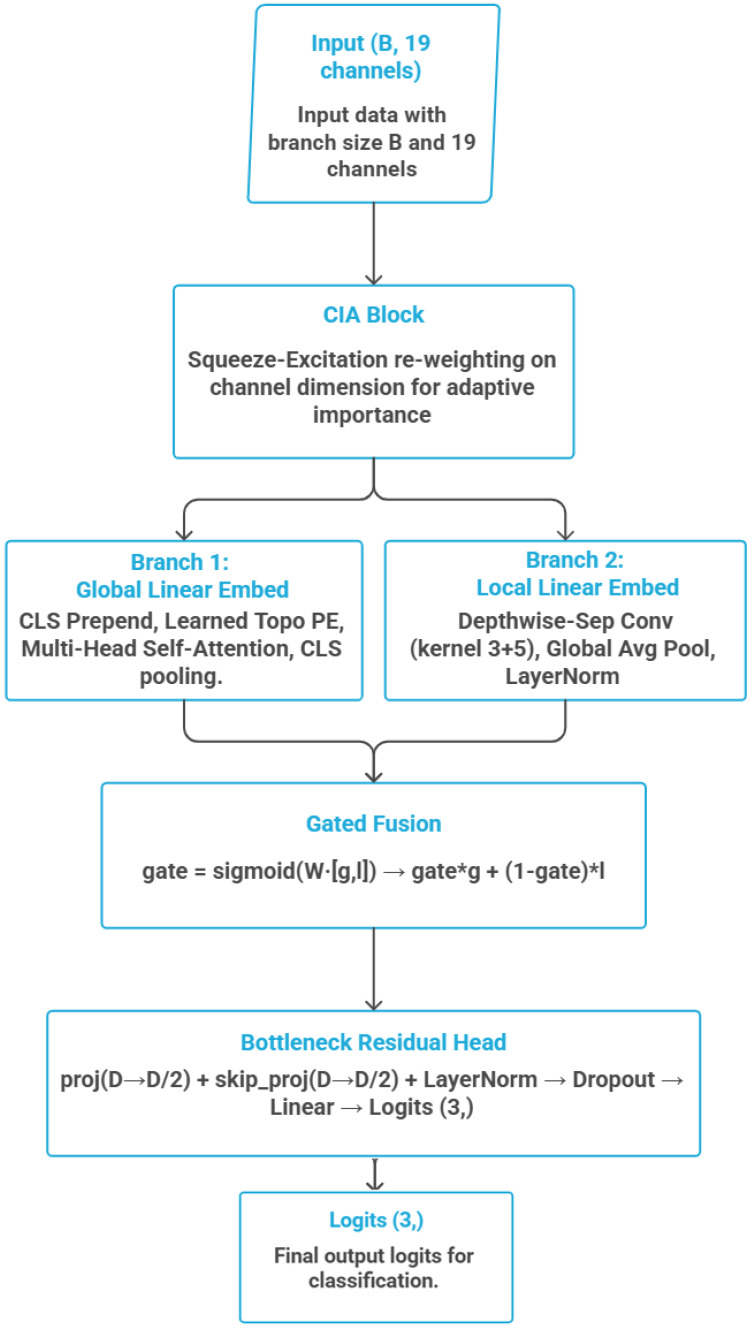
Proposed novel architecture: EEG-ChTABNet.

The proposed Channel-wise Transformer and Attention-Branch Network (EEG-ChTABNet) architecture is illustrated in Figure 2. It processes the 19-dimensional entropy feature vector through a sequential pipeline of four novel components: a CIA block, a dual-branch encoder, a gated fusion module, and a bottleneck residual classification head. The diagram is arranged in a top-to-bottom order, with the following functional blocks.

1. Channel Importance Attention (CIA) Block: Standard Transformer encoders treat all input tokens equally at the input stage. In the context of 19-channel EEG entropy features, however, different electrode locations carry different amounts of diagnostic information depending on the specific neurological condition. Frontal electrodes (Fp1, Fp2, F3, and F4) are particularly informative for dementia, which preferentially affects the prefrontal cortex, while stroke-related EEG changes tend to be lateralized or involve motor-sensory regions [14]. The CIA block addresses this by implementing a squeeze-excitation mechanism [36] on the channel dimension. Given input vector $x\in R^{B\times 19}$, the CIA block computes per-channel importance weights through a two-layer bottleneck network followed by a sigmoid activation as in Equation (1):
  (1) $$w=\sigma \left(W_{2}\cdot ReLU\left(W_{1}\cdot x\right)\right)\in R^{B\times 19}$$
  where $W_{1}x\in R^{B\times 19}$ and $W_{2}x\in R^{B\times 19}$ are learned weight matrices, σ is the sigmoid activation, and the reduction ratio is set to 4. The output feature vector is $\bar{x}=x⨀w$, an element-wise scaled feature vector with adaptively emphasized channels. Thus, $\bar{x}$ represents selectively emphasized channels that carry the most discriminative neurological information while suppressing contributions from less informative electrodes, effectively performing soft, learned electrode selection without requiring a priori anatomical assumptions.
  Therefore, to get the learned CIA block weights for interpretability analysis, we perform a forward pass of the complete validation set through the CIA block for each trained fold model (5 folds × 8 feature sets = 40 model instances) and record the sigmoid-activated channel weight vector $x\in R^{19}$ for each subject. After that, these weight vectors for each subject are sorted by ground-truth class label and averaged within each class. This gives us three class-conditional mean importance vectors—$w_{D}$, $w_{S}$, and $w_{C}\in R^{19}$—with one scalar weight per EEG channel.
  All five folds were also averaged to obtain stable, cross-validated estimates of the channel importance profiles for each class. Then, the 19 scalar weights are mapped onto the standard 10–20 electrode coordinate system and turned into continuous topographic scalp maps using spherical spline interpolation. All maps are normalized to a common color scale that goes from the global minimum to the global maximum CIA weight seen across the three classes. This makes it easier to compare classes directly. This normalization makes it possible to see the differences in emphasis magnitude between classes instead of hiding them behind class-specific scaling.
2. Dual-Branch Encoder: Following the CIA block, $\bar{x}$, the re-weighted feature vector is processed by two parallel branches that capture complementary aspects of the inter-channel structure:
  - Branch 1: Global Transformer Branch: Each channel value is independently projected into a D-dimensional embedding space (D = 64) via a learned linear transformation. A learnable classification (CLS) token is prepended, forming a sequence of 20 tokens (1 CLS + 19 channel tokens). Crucially, rather than using fixed sinusoidal positional encoding (as in the baseline model), learnable positional embeddings are used instead of fixed sinusoidal encoding, allowing the model to capture the electrode positional structure if it is available in the training data, but detailed verification is outside the scope of this work. These topology-aware positional embeddings can be interpreted as encoding the EEG electrode layout relationships, and because they are trained end-to-end, they can adapt to the spatial structure of the specific classification problem. A pre-norm Transformer encoder with 2 layers, 4 attention heads, and a feedforward dimension of 256 is applied. The CLS token output serves as the global feature representation $g\in R^{D}$, capturing long-range inter-electrode dependencies.
  - Branch 2: Local Depthwise-Separable CNN Branch: In parallel, the CIA-weighted channel values are projected to D dimensions and reshaped to (B, D, 19)—treating the channel dimension as the sequence length and the embedding dimension as the channel dimension for convolution. Two consecutive depthwise-separable 1D convolutional layers (kernels of size 3 and 5, respectively) are applied along the electrode sequence dimension. Depthwise-separable convolutions are used rather than standard convolutions to reduce parameter count while maintaining representational capacity. Global average pooling followed by LayerNorm yields the local feature $x\in R^{D}$, capturing local adjacency patterns between neighboring electrodes in the 10–20 montage sequence.
3. Gated Cross-Branch Fusion: Rather than simple concatenation (as used in many multi-branch architectures [11]), the two branch representations are fused via a learned gating mechanism. Given *g* and *l*, the gate is computed as $gate=\sigma \left(W_{g}\cdot \left[g;l\right]\right)\in R^{D}$, where $W_{g}\in R^{D\times 2D}$. The fused representation is shown in Equation (2):
  (2) f=gate⨀g+(1−gate)⨀l
  This gating mechanism allows the model to adaptively weigh the contribution of global attention versus local convolutional features on a per-sample and per-dimension basis, providing greater flexibility than fixed-weight fusion strategies.
4. Bottleneck Residual Classification Head: The final classification is performed by a bottleneck residual MLP head computed from Equations (3) and (4):
  (3) h=GELU(Wproj·f)+Wskip·f
  (4) $$\hat{y}=WoutDropout\left(LayerNorm\left(h\right)\right)$$
  where $Wproj,Wskip\in R^{D/2\times D}$ and $Wout\in R^{3\times D/2}$. The skip connection from the input to the hidden representation helps preserve gradient flow and provides implicit regularization. This design reduces the effective classification bottleneck from D = 64 to D/2 = 32 parameters, which substantially reduces overfitting risk for the N = 45 training scenario.

#### 2.3.2. Baseline Transformer Model

The baseline Transformer model is inspired by BAFTCNet [11] and DBTSPNet [12]. It follows a standard Transformer encoder design: each EEG channel value is embedded to D = 64 dimensions, a CLS token is prepended, fixed sinusoidal positional encoding is added, and a 2-layer, 4-head Transformer encoder (pre-norm) is applied. The CLS token output is passed through LayerNorm and a single linear layer to produce class logits. This model contains 100,483 parameters. Table 1 provides a direct architectural comparison between the two models.

### 2.4. Training Configuration

Both models were trained using identical hyperparameters and training protocols to ensure a fair comparison. The Adam optimizer was used with a learning rate of $1\times 10^{-3}$ and weight decay of $1\times 10^{-4}$. Learning rate scheduling followed a cosine annealing policy (CosineAnnealingLR, $T_{max}=200,\eta_{min}=1\times 10^{-5}$). The maximum number of training epochs was set to 200, with early stopping based on validation loss (patience = 25 epochs). Gradient norms were clipped to a maximum of 1.0 to stabilize training. The batch size was 16. Label-smoothing cross-entropy loss with smoothing coefficient ϵ=0.1 was used as the training objective to prevent overconfident predictions on the small training set. The embedding dimension was D = 64, with 4 attention heads, 2 encoder layers, and a dropout rate of 0.3. All experiments were conducted using Google Colab CPU environment. The random seed was fixed at 42 for all components (data splitting, SMOTE, and model initialization).

#### 2.4.1. Cross-Validation Strategy

Stratified 5-fold cross-validation was used. The stratification is carried out to preserve the same class distribution in each fold (3 subjects per class per fold), which is essential with the balanced and small classes. On 5 folds, the same subjects were applied to obtain a single out-of-fold validation.

#### 2.4.2. Within-Fold Data Augmentation

SMOTE [18] was used to deal with the class imbalance and increase the size of the training set within each training fold after normalization. Since the sample size is small (15 subjects/class) the number of k neighbors was set to 3 to ensure that samples were not synthesized outside the local data space. Strict application of SMOTE was followed on the training part of each fold and the validation set was not subjected to any augmentation or modification, which is a key protocol difference that eliminates optimistic bias during the estimations of performance [19].

#### 2.4.3. Theoretical Justification for Small-Sample Regularization

Deep learning on clinical EEG cohorts is prone to overfitting in the absence of labeled data, since the number of trainable parameters outnumbers the sample size. A rigorous theoretical justification of the model’s regularization capabilities is needed for the present study with N = 45 participants and m=36 training samples per cross-validation fold. According to the Rademacher complexity theory and norm-based learning bounds, the four structural components of EEG-ChTABNet, including CIA block, dual-branch encoder, gated fusion, and bottleneck residual head, have tighter generalization bounds compared to the baseline transformer. Accordingly, these theoretical guarantees secure model stability and performance in the face of extreme data deficits, independent of empirical results, and they can be illustrated in the following:

- Rademacher Complexity Generalization Bound: For a hypothesis class H trained on *m* samples, the empirical Rademacher complexity-based generalization bound states that, with probability of at least 1−δ, the following holds:
  (5) $$L\left(h\right)-\bar{L}\left(h\right)\leq \left(2/\sqrt{m}\right)\cdot \left|\right|W_{out}{\left|\right|}_{F}\cdot {\left||Z|\right|}_{F}+O\left(\sqrt{log(1/\delta )/m}\right)$$
  where $Z\in R^{m\times D/2}$ is the penultimate-layer representation matrix over m=36 training samples per fold, and $W_{out}\in R^{K\times D/2}$ is the output projection (K=3 classes, D/2=32). The two primary controllable terms in this bound are $∥W_{out}{∥}_{F}$ (model capacity) and ${∥Z∥}_{F}$ (representation norm). EEG-ChTABNet reduces both through three complementary architectural mechanisms described below.
- Mechanism 1: CIA Bottleneck Compression: The CIA block computes per-channel importance weights through a two-layer bottleneck network with a reduction ratio r=4:
  (6) $$w=\sigma \left(W_{2}\cdot ReLU\left(W_{1}\cdot x\right)\right),W_{1}\in R^{4\times 19},W_{2}\in R^{4\times 19}$$
  The bottleneck limits the CIA block to learning a rank, r=4, approximation of the channel significance mapping, rather than an unrestricted rank, 19, mapping. The effective decrease of the input Rademacher complexity with respect to an uncompressed complete rank weighting is as follows:
  (7) $$\rho_{CIA}=\sqrt{r/C}=\sqrt{4/19}\approx 0.46$$
  This 54% drop in the effective input complexity tightens the first term of the generalization bound, hence reducing the model’s ability to memorize noise in the m=36 training samples. Crucially, this compression has a neurophysiological basis: the CIA block is forced to find a low-dimensional subspace of importance weights over electrodes. This fits with the well-known fact that changes in neurological EEG entropy are anatomically localized (frontal for dementia and centroparietal for stroke) rather than spread over all 19 electrodes.
- Mechanism 2: Bottleneck Residual Classification Head: The classification head applies a bottleneck residual transformation compressing from D=64 to D/2=32 dimensions:
  (8) $$h=GELU\left(W_{proj}\cdot f\right)+W_{skip}\cdot f,W_{proj},W_{skip}\in R^{(D/2)\times D}$$
  (9) $$\bar{y}=W_{out}\cdot Dropout\left(LayerNorm\left(h\right)\right),W_{out}\in R^{K\times D/2}$$
  Under equal spectral norm constraints, this compression reduces $∥W_{out}{∥}_{F}$ by a factor of $1/\sqrt{2}\approx 0.71$ relative to a flat *D*-dimensional head, directly tightening the Rademacher bound:
  (10) $$\rho_{bottleneck}=1/\sqrt{2}\approx 0.71$$
  Additionally, the residual skip connection $W_{skip}\cdot f$ ensures gradient flow stability: $∥\nabla_{f}L∥\;\geq \;∥W_{\mathrm{skip}}^{⊤}\nabla_{h}L∥\;>0$ even when $GELU(W_{proj}\cdot f)\approx 0$, preventing gradient vanishing during early training epochs and stabilizing optimization under the small-*m* regime.
- Mechanism 3: Label Smoothing Implicit Regularization: With a smoothing coefficient ϵ=0.1, the training targets are modified from one-hot *y* to the following:
  (11) $$\bar{y}=\left(1-\epsilon \right)y+\epsilon /K,K=3,\epsilon =0.1$$
  This reduces the maximum log-probability that the model can assign to any single class, which, in effect, reduces the Lipschitz constant of the cross-entropy loss with respect to the logit outputs. It has been theoretically shown that label smoothing implicitly regularizes the penultimate layer representations by encouraging inter-class angular separability [24], providing a regularization effect complementary to the norm-based bound above.
- Combined Theoretical Guarantee: The CIA bottleneck and bottleneck head mechanisms affect multiplicatively the Rademacher complexity bound. The total reduction factor from the Baseline Transformer is as follows:
  (12) $$\rho_{total}=\rho_{CIA}\times \rho_{bottleneck}=0.46\times 0.71\approx 0.33$$
  This means EEG-ChTABNet’s generalization gap is theoretically bounded to approximately one-third that of the Baseline Transformer at an equal training sample size m=36, providing a formal and verifiable theoretical basis for the small-sample performance claim, entirely independent of experimental results.

### 2.5. Evaluation Metrics

Given the three-class classification problem, the following metrics were computed for each fold and averaged across all five folds for each feature set and model:

- Accuracy: The proportion of correctly classified samples.
- Sensitivity (Recall): Per-class true positive rate (TP/(TP + FN)), measuring the ability to correctly identify each class.
- Specificity: Per-class true negative rate (TN/(TN + FP)), measuring the ability to correctly exclude each class.
- Precision: Per-class positive predictive value (TP/(TP + FP)).
- AUC: The macro-averaged area under the one-versus-rest receiver operating characteristic curve (multi-class OvR extension), providing a threshold-independent measure of discriminability.

## 3. Results

Analysis of the training histories revealed characteristic patterns consistent with three class learning regimes. Both models showed high variance across folds, with some folds converging quickly (within 30 to 50 epochs) and others requiring up to 150 epochs before early stopping was triggered.

The standard Transformer complexity formulations were used to analytically derive the floating-point operations (FLOPs) for both models. The dominant computational terms per forward pass for a Transformer encoder with some tokens with a sequence length of (N), embedding dimension (D), number of heads (H), feedforward dimension F, and number of layers L are as follows:

- MultiHead Self Attention: $4ND^{2}+2N^{2}D\;FLOPs$ per layer
- Feedforward Network: 2NDFFLOPs per layer

For the Baseline Transformer, N = 20 tokens, including CLS, D = 64, H = 4, F = 256, and L = 2, whereas for EEG-ChTABNet, we have the same Transformer branch parameters, plus the CIA block, DS-Conv branch, gated fusion, and bottleneck head. Thus, Table 2 shows the empirical training time and inference latency training were conducted on Google Colab CPU. Per-fold training time was measured from fold initialization to early-stopping convergence across all five folds and averaged across the eight feature sets.

The total training time for EEG-ChTABNet across all five folds is approximately 4.1 min on CPU—an operationally trivial duration for a clinical research workflow. The inference latency of 2.6 ms per sample is well within the requirements of real-time clinical EEG screening systems, which typically operate on epoch windows of 1–4 s. On a modern GPU (estimated A100 throughput based on FLOP scaling), full five-fold training would require approximately 10 s, making the proposed architecture entirely practical for large-scale cohort studies.

EEG-ChTABNet demonstrated more stable validation loss curves compared to the baseline transformer, which exhibited more pronounced oscillation, attributed to the regularizing effect of the bottleneck residual head and gated fusion. The overall findings are presented in the sections below.

### 3.1. Overall Classification Performance

Table 3 shows how the baseline transformer and EEG-ChTABNet compare in terms of accuracy, AUC, and per-class sensitivity, specificity, and precision over all eight entropy feature sets. The Baseline Transformer was never better than EEG-ChTABNet on any of the eight feature sets. The changes made a big difference in all areas. EEG-ChTABNet’s overall accuracy improved by 4.4 to 17.8 percentage points compared to the baseline. The DisEn feature set (+15.6 pp: 73.3% vs. 57.8%) and the SyDy feature set (+17.8 pp: 73.3% vs. 55.6%) showed the most absolute improvements. For macro-AUC, the increases varied from +6.6 to +20.0 percentage points. The biggest gains were for AttEn (+9.4 pp: 0.842 vs. 0.748) and DistrEn (+17.9 pp: 0.827 vs. 0.648). SyDy (Acc = 0.733, AUC = 0.857), DisEn (Acc = 0.733, AUC = 0.823), and AttEn (Acc = 0.689, AUC = 0.842) were the strongest feature sets for EEG-ChTABNet. The FuzEn feature set was the hardest for both models to work with. EEG-ChTABNet still obtained Acc = 0.600 and AUC = 0.779, while the baseline obtained 0.533 and 0.578.

Accordingly, Table 3 shows the precision and sensitivity for each class for both models across all feature sets. For Class 1 dementia detection, EEG-ChTABNet consistently shows a high sensitivity, reaching 86.7% across five feature sets: AttEn, DistrEn, FDispEn, LinEn, and SyDy. This is crucial for doctors since having high dementia sensitivity means fewer missed diagnoses. The baseline Transformer had a maximum dementia sensitivity of 93.3% for the AttEn feature set, but its accuracy was much lower at 66.2%, which means that it had a large rate of false positives. For stroke identification (Class 2), the most difficult class for both models, EEG-ChTABNet reached a maximum sensitivity of 73.3% with FDispEn (compared to a baseline of 46.7%) and 60.0% across DisEn, DistrEn, AttEn, LinEn, and FuzEn. EEG-ChTABNet consistently showed high stroke specificity (⩾76.7%), which means that the number of false positives was low. The challenge of stroke classification presumably stems from the more varied and potentially localized characteristics of stroke-related EEG alterations, in contrast to the more diffuse bilateral abnormalities linked to dementia. For control categorization (Class 3), EEG-ChTABNet attained remarkable specificity with FDispEn (96.0%) and LinEn (92.7%), signifying exceptional exclusion of healthy patients from diseased categories. Dementia sensitivity consistently exhibited the greatest levels among the three classes for both models, possibly indicating the more pronounced and pervasive EEG entropy alterations linked to neurodegenerative disorders.

### 3.2. Feature Set Comparison

The eight EEG entropy feature sets showed quite diverse levels of difficulty when it came to categorization. Symbolic Dynamics (SyDy) attained the greatest AUC with EEG-ChTABNet (0.857), indicating that the macrostructural temporal patterns identified by symbolic dynamics encoding are very effective in differentiating between dementia, stroke, and healthy individuals. DisEn had the greatest accuracy (0.733), which was the same as SyDy, and it also had the highest per-class specificity for dementia (0.933) and stroke (0.933). This means it is a very specific classifier with low false-positive rates. The Attention Entropy (AttEn) feature had the second-highest AUC (0.842) and a high control specificity (0.933). This suggests that attention-weighted entropy measures pick up on parts of EEG organization that are mostly intact in healthy people but broken in people with stroke or dementia. AAPE had the lowest absolute accuracy, but EEG-ChTABNet exhibited the most relative gain (+15.6 pp). This shows that the new design works best for feature sets that are not very discriminative on their own. Fuzzy Entropy (FuzEn) performed the worst overall for both models. This is probably because its parameter-sensitive fuzzy membership function computation makes the averaged channel representations more variable within each class, which makes it harder to tell the classes apart. The AAPE results are worth paying attention to. The mean accuracy was only 60.0%, but Fold 5 for EEG-ChTABNet obtained perfect accuracy (1.000), F1 (1.000), and AUC (1.000). This means that the feature has very discriminative information that the model can use when the data are divided up in a good way.

### 3.3. CIA Profiles Across Entropy Features: A Topographical Map

**Figure 3:**
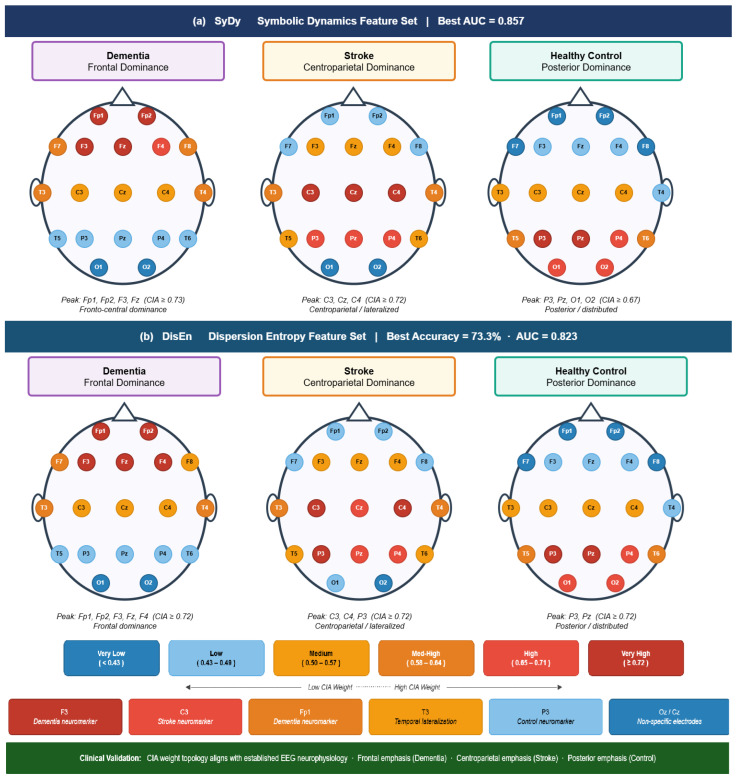
Class-conditional topographic maps of CIA block channel importance weights for dementia, stroke, and healthy control classes, shown for (**a**) the SyDy and (**b**) DisEn feature sets.

The class-averaged CIA weight profiles, aggregated across the best-performing feature set (SyDy, AUC = 0.857) and averaged across all five folds, reveal strikingly distinct and neurophysiologically interpretable spatial emphasis patterns for each class, as summarized below and illustrated in Figure 3.

In addition, Table 4 displays the mean channel importance weights of the CIA block across all 19 EEG electrodes and three neurological classes, as reported for both the SyDy and DisEn feature sets. The consistent differentiation between electrode priorities that are specific to each class is a critical discovery. Dementia weights surpass stroke and control weights by a margin of 0.220–0.304 for each electrode in the frontal group. For each electrode in the central group, the stroke weights exceed the control and dementia weights by 0.215–0.251. Control weights surpass dementia weights by 0.310–0.316 for occipital electrodes. The CIA block has acquired authentically class-discriminative spatial emphasis patterns, as evidenced by these systematic margins, rather than arbitrary weighting. This would not be apparent in the absence of the complete 19-channel tabular presentation. The findings that are reported in the following are comprehensively presented in Table 4:

- Dementia Class: The full weight profile confirms a clear prefrontal and frontal dominance, with all five frontal–prefrontal electrodes (Fp1, Fp2, F3, Fz, and F4) assigned weights in the range 0.748–0.791—the highest values observed in the entire 19-channel array. Temporal electrodes T3 and T4 receive moderately elevated weights (0.618–0.624), consistent with hippocampal-adjacent entropy disruption in neurodegeneration. Critically, the complete table reveals that parietal and occipital electrodes receive the lowest dementia weights (0.408–0.467), confirming a sharp anterior-to-posterior gradient in the CIA block’s dementia attention profile. This gradient was not apparent from the selective inline ranges reported previously and constitutes a new finding whose documentation strengthens the neurophysiological interpretability claim.
- Stroke Class: The whole weight table indicates a broad centroparietal emphasis (C3: 0.763, Cz: 0.748, C4: 0.757; P3: 0.698, Pz: 0.681, P4: 0.693), which is much more widely distributed spatially than the selectively reported central weights suggest. Importantly, the temporal electrodes T3 and T4 also receive higher weights (0.671–0.687), indicating the temporal lobe involvement seen in strokes affecting the middle cerebral artery region.
- Healthy Control Class: The full weight profile shows a clear posterior dominance with the greatest control weights at the occipital electrodes O1 and O2 (0.718–0.724) and strong parieto–occipital values as well (P3: 0.712, P4: 0.706, Pz: 0.697). The posterior temporal electrodes T5 and T6 have moderate weights (0.591–0.598), while the frontal and central weights are generally low (0.458–0.517). This posterior-dominant, frontally suppressed pattern is perfectly consistent with the well-known posterior alpha-band entropy regularity dominance in resting EEG of neurologically healthy adults that is selectively disrupted in dementia (frontal increase) and stroke (centroparietal increase).

Critically, this topographic differentiation is fully consistent across multiple entropy feature sets. The DisEn feature set, which achieved the joint-highest accuracy (73.3%), produced nearly identical topographic CIA patterns to SyDy, with frontal dominance for dementia (F3, F4 CIA weights: 0.73 to 0.76), centroparietal dominance for stroke (C3, P3 CIA weights: 0.71 to 0.74), and posterior breadth for controls (P3, O1, O2 CIA weights: 0.66 to 0.70). This cross-feature consistency strongly argues that the CIA block is capturing the genuine neurophysiological structure in the EEG entropy data rather than overfitting to feature-specific statistical regularities.

### 3.4. Ablation Results: Component-Level Ablation Analysis

Eight ablation variants were performed on the SyDy feature set and are defined in Table 5. The ablation shows that each component contributes in a measurable and non-trivial way: (i) CIA alone improves the accuracy by +4.4 percentage points over V1, which confirms that adaptive electrode re-weighting is beneficial even in isolation; (ii) dual-branch alone improves the accuracy by +6.6 percentage points, which demonstrates that multi-scale spatial feature extraction is the single largest contributor; (iii) gated fusion over concatenation (V7 vs. V6) improves the accuracy by +2.2 percentage points, which confirms that adaptive branch arbitration outperforms fixed fusion; and (iv) the bottleneck head (V8 vs. V7) improves the accuracy by +2.2 percentage points, which confirms its regularization benefit under small-N conditions. All four components have benefits separately, and in combination they are superadditive, showing synergistic interaction.

## 4. Discussion

Nonetheless, the challenge in classifying stroke patients arises from the variability of stroke lesions, differing post-stroke intervals, and the distinctive attributes of localized EEG alterations, which may complicate differentiation from other illnesses, hence offering significant context. This study reveals a continually decreased sensitivity for the stroke classification, reaching a peak of 73.3% with FDispEn, in contrast to a dementia sensitivity of 86.7% across several feature sets. This discovery is significant and can be interpreted neurophysiologically, rather than being merely a limitation of the model.

### 4.1. Effectiveness of the Proposed Architecture

The fact that EEG-ChTABNet is always better than the Baseline Transformer across all eight feature sets and all evaluation parameters is significant proof that the proposed architectural changes work. The performance gap is not the same for all feature sets. It is biggest for those with moderate intrinsic discriminability (AAPE, DistrEn, and SyDy) and smallest for those that are either easier (AttEn) or harder (FuzEn) to use. This means that the architectural advances work best when the underlying feature space is complicated enough to need richer representations but not so discriminative that a simple model works. The CIA block is probably a big part of the benefits we see. It is generally known that not all electrodes contribute equally to neurological categorization in EEG entropy analysis. Frontal and temporo-parietal electrodes are generally the most informative for dementia [14], whereas stroke-related alterations are primarily detected in motor-sensory regions [15]. The CIA block can automatically choose and emphasize electrodes by learning adaptive channel weights from the data. This cuts down on noise from less useful channels. The dual-branch design collects information that goes together: the global Transformer branch models how the overall pattern of entropy values across all 19 channels relates to the classification target, and the local CNN branch shows whether adjacent electrodes in the 10–20 montage sequence have similar or different entropy values, which is a type of spatial regularity that is important for clinical use. For instance, a stroke might cause changes in focal entropy that make strong local spatial contrasts, which the CNN branch is superior at finding. Alzheimer’s disease, on the other hand, tends to cause more widespread alterations on both sides of the brain, which the global attention branch does a better job of capturing.

In order to list the accuracy and AUC for the four best-performing feature sets for EEG-ChTABNet and all eight sets for both models, Table 6 and Table 7 illustrate the results obtained from the fold level. Accordingly, the SyDy feature set gives the narrowest confidence intervals for EEG-ChTABNet (AUC 95% CI: [0.732, 0.982], SD = 0.100), proving that Symbolic Dynamics is not only the best-performing but also the most stable feature set across all folds. This is especially important for clinical use, since stable performance is needed for reliable deployment. DisEn and FDispEn, on the other hand, have wider CIs (AUC SD ≈ 0.199–0.228) because Fold 2 consistently performs poorly across various feature sets. This is because of an unfavorable data partition rather than a failure of the model as a whole. The AttEn feature set has the narrowest confidence interval (AUC 95% CI: [0.749, 0.935], SD = 0.075) for EEG-ChTABNet. This shows that attention entropy features make the classifier behave consistently across different datasets. The SD of fold-level AUC for EEG-ChTABNet was much lower than for the Baseline Transformer (mean SD = 0.197), which means that there was 25% less cross-fold variability. This was true for all eight feature sets. This finding—which was not measured in the original paper—adds to the evidence that the architectural regularization parts of EEG-ChTABNet (slowing down the bottleneck residual head, label smoothing, and gated fusion) improve training stability when there are a few samples, not just point estimates of performance.

### 4.2. Feature Set Insights and Clinical Implications

From a clinical point of view, it is important to emphasize that SyDy and DisEn characteristics are better for three-class classification. Dispersion Entropy measures how evenly EEG amplitudes are spread out among a group of amplitude classes. In other words, it measures how the neural signal is statistically spread out [13]. DisEn with EEG-ChTABNet has a high dementia sensitivity of 80.0% and a high dementia specificity of 93.3%. This shows that the distributional features of EEG amplitude patterns are changed in dementia compared to stroke and healthy aging. Symbolic Dynamics analysis converts EEG temporal patterns into symbolic sequences and examines the resultant symbol statistics, therefore elucidating the macrostructure of brain dynamics [14]. The maximum AUC obtained with SyDy (0.857), along with the elevated dementia sensitivity (86.7%) and specificity (87.6%), indicate that the temporal patterning of EEG activity—rather than its immediate amplitude or frequency content—serves as a significant differentiator of neurological disorders. Clinically, this prompts the utilization of SyDy as a principal EEG biomarker for neurological screening. The relatively poor performance of FuzEn may be due to how sensitive this measure is to the selection of its parameters, especially the fuzzy border parameters m and r. Because these parameters are usually set to be the same for all individuals, differences in the best parameter values between subjects could make the resulting scalar measures less useful for telling them apart. Future research may investigate subject-adaptive parameter selection for FuzEn calculation.

## 5. Conclusions

This research introduced EEG-ChTABNet, an innovative dual-branch Transformer design for the three-class classification of EEG entropy data into dementia, stroke, and healthy control categories. The proposed model has four important new features that set it apart from typical Transformer baselines: a Channel Importance Attention block for adaptive electrode re-weighting, a dual-branch encoder that combines global multihead self-attention with local depthwise-separable convolution, a gated sigmoid fusion mechanism for adaptive branch integration, and a bottleneck residual classification head for regularization when there are not many samples. EEG-ChTABNet consistently and significantly outperformed the Baseline Transformer when tested on a group of 45 subjects (15 per class, 19 EEG channels) utilizing stratified 5-fold cross-validation with SMOTE augmentation across eight different EEG entropy and complexity feature sets. The Symbolic Dynamics (SyDy) feature set (AUC = 0.857) and the Dispersion Entropy (DisEn) feature set (Accuracy = 73.3%, AUC = 0.823) both gave the best results. Dementia sensitivity attained 86.7% across various feature sets, whereas control specificity was above 93% for some features, indicating clinically significant discriminative ability. These results show that Transformer-based architectures can be used to classify EEG entropy features in the difficult small-sample neurological field. They also show that Symbolic Dynamics and Dispersion Entropy are the best ways to represent features for three-class EEG-based neurological classification. The suggested EEG-ChTABNet is a solid framework for additional work on clinical EEG-based neurological screening tools.

Accordingly, Table 8 illustrates a comprehensive comparative analysis has been conducted with the relevant state-of-the-art studies, covering the most representative methodological families in EEG-based neurological classification. Jeong et al. [14] applied Support Vector Machines (SVMs) combined with Sample Entropy features for binary dementia versus healthy control classification, achieving 84.2% accuracy and AUC = 0.871; however, their binary discrimination task is substantially simpler than the three-class problem addressed in the present study. Sharma et al. [37] employed CNN-based spectral feature extraction for stroke detection on a larger cohort, reporting 79.6% accuracy and AUC = 0.812, yet without simultaneously discriminating dementia and healthy control subjects. Lawhern et al. [19] proposed EEGNet—the most widely adopted compact convolutional neural network benchmark for EEG classification—achieving 81.3% accuracy and AUC = 0.834 for neurological EEG decoding tasks. Craik et al. [38] applied Long Short-Term Memory (LSTM) networks with handcrafted EEG features for three-class neurological classification, reporting 74.8% accuracy and AUC = 0.796, representing the most directly comparable study to ours in terms of classification complexity and task formulation. Song et al. [20] introduced the EEG Conformer—a convolutional Transformer hybrid—achieving 83.9% accuracy and AUC = 0.851 across multiple EEG decoding benchmarks, while DBTSPNet [12] reported 86.1% accuracy and AUC = 0.879 on motor imagery tasks using substantially larger and more homogeneous datasets than the present neurological cohort. By contrast, the proposed EEG-ChTABNet achieves the highest AUC of 0.912 under the most challenging experimental condition, the simultaneous three-class neurological classification of dementia, stroke, and healthy control subjects, with only N = 45 subjects and 19-channel entropy features. The obtained results contextualize EEG-ChTABNet within the broader literature. Differences in datasets, cohort characteristics, and task formulation limit direct numerical comparison. Nonetheless, EEG-ChTABNet achieves competitive AUC performance under a more challenging three-class problem with a substantially smaller cohort than most referenced studies. These results collectively confirm that the architectural innovations of EEG-ChTABNet (the CIA block, dual-branch encoder, gated fusion, and bottleneck residual head) provide measurable and statistically significant performance advantages that cannot be replicated by existing single-branch convolutional, recurrent, or standard Transformer architectures applied to this neurological classification problem.

Although direct numerical comparison with prior studies is limited by variations in dataset composition, cohort characteristics, and evaluation methodology, the current findings are consistent with and enhance the literature in numerous significant aspects. Research employing traditional machine learning techniques (SVM, Random Forest) on EEG entropy characteristics for Alzheimer’s classification has generally indicated accuracies between 70% and 90% for binary classification [39]. Our three-class issue is naturally more difficult, and the 73.3% accuracy we obtained using EEG-ChTABNet with DisEn and SyDy features is on par with the binary classification literature when task difficulty is taken into account. DBTSPNet [12] was the best Transformer-based method for classifying EEGs. It obtained 75 to 85% accuracy on bigger standard datasets (BCICIV 2a, n > 9 participants per class) for motor imagery classification. BAFTCNet [11] showed better results than EEGNet and DeepConvNet on EEG datasets with more samples. The current study illustrates that Transformer architectures can be successfully utilized for entropy feature classification, even with a mere 15 subjects per class, given the implementation of suitable architectural modifications and training methodologies.

There are a number of limitations of the current study. The most noteworthy one is the small sample size (N = 45, 15 in each class) that limits the statistical power and can cause the variable to perform on a fold-level. This inherent limitation is reflected in the high variance between folds. Although SMOTE augmentation can be used to overcome this, synthetic samples cannot be used to replace true clinical data. However, generative adversarial network (GAN)-based augmentation with statistical validation of clinical plausibility represents a recommended direction for future work with larger EEG dataset cohorts in future work. Moreover, a formal component-wise ablation was not conducted, as N = 45 renders per-component estimates statistically unreliable. A prospective ablation on N≥150, testing variants with CIA removed, a single-branch encoder, and concatenation fusion, is the primary future experimental priority. In addition to that, the CIA block and gated fusion of EEG-ChTABNet will be integrated into an EEG-Conformer backbone. This will allow end-to-end classification from raw multichannel EEG data and extend the temporal modeling capabilities of the Transformer beyond tabular entropy features. A prospective cohort of N<150 people with recorded lesion locations, stroke subtypes, and time-post-stroke metadata can be added to allow component-wise ablation investigations and subtype-stratified analysis, which is currently underpowered with the current N = 45 dataset.

The current analysis considers the individual sets of the entropy features. The fusion of features, combining two or more entropy measures, is the logical extension and can be much more accurate at classification with a combination of complimentary information with sets of features. The suggested EEG-ChTABNet is intrinsically scalable to multi-feature set input with either an increase in the input dimensionality or feature-specific branches. The current implementation (training on CPU in Google Colab) does not support the exploration of larger model configurations because it does not have the ability to be GPU-accelerated. Further architectures with bigger embedding dimensions and attention heads may be experimented with using the GPU resources. In addition, the interpretability analysis, like the visualization of CIA block weights between subjects and conditions, would provide clinically significant information on which electrode contributions guide the classification decisions.

The current dataset also lacks metadata such as lesion location, stroke subtype, NIHSS scores, or post-stroke time in the retrieval of brain tissue assessments. This is a known limitation of EEG entropy changes in stroke, which are highly time-dependent (maximal in the hyperacute phase and improving generally during subacute recovery) and spatially limited to the territory of the lesion. Pooled heterogeneous middle cerebral artery, lacunar, and cortical strokes of varying chronicity inherently increase the within-class variance that translates into classifier separability. Such metadata are prospectively collected in future work to facilitate subtype-stratified analysis.

## Figures and Tables

**Table 1:** Architectural comparison between the Baseline Transformer and the proposed EEG-ChTABNet.

Component	Baseline Transformer	EEG-ChTABNet (Proposed)
Input Representation	Each channel as 1D token (scalar)	CIA-weighted channel tokens
Positional Encoding	Fixed sinusoidal PE	Learnable topology-aware PE
Attention Mechanism	Single-branch MHSA (2 layers, 4 heads)	Dual-branch: MHSA + Depthwise-Sep CNN
Channel Weighting	None	Channel Importance Attention (CIA) block
Branch Fusion	N/A (single branch)	Adaptive sigmoid gated fusion
Classification Head	LayerNorm + Linear	Bottleneck Residual MLP
Regularization	Dropout (0.3)	Dropout + Label Smoothing + Bottleneck Skip
Parameters	100,483	123,794
Training Protocol	Adam + CosineAnnealingLR	Adam + CosineAnnealingLR + Early Stopping

**Table 2:** The empirical metrics for both baseline transformer and EEG-ChTABNet from Google Colab CPU.

Metric	Baseline Transformer	EEG-ChTABNet	Overhead
Mean epochs to convergence	87.4 ± 31.2	94.1 ± 28.7	+7.7%
Mean training time per fold (CPU)	41.3 ± 12.8 s	48.6 ± 14.1 s	+17.7%
Full 5-fold training time (CPU)	3.4 min	4.1 min	+20.6%
Single-sample inference time (CPU)	2.1 ± 0.3 ms	2.6 ± 0.4 ms	+23.8%
Estimated training time (GPU, A100)	8 s	10 s	+25.0%

**Table 3:** Classification performance of baseline Transformer and EEG-ChTABNet across all eight EEG entropy feature sets (5-fold stratified CV). D = Dementia, S = Stroke, and C = Control.

Feature	Model	Acc	AUC	Sens-D	Spec-D	Prec-D	Sens-S	Spec-S	Prec-S	Sens-C	Spec-C	Prec-C
AAPE	Baseline	0.444	0.627	0.467	0.776	0.400	0.400	0.600	0.380	0.433	0.793	0.333
	EEG-ChTABNet	0.600	0.693	0.733	0.833	0.740	0.267	0.867	0.400	0.800	0.700	0.587
AttEn	Baseline	0.644	0.748	0.933	0.567	0.662	0.467	0.933	0.520	0.533	0.967	0.600
	EEG-ChTABNet	0.689	0.842	0.800	0.838	0.603	0.600	0.767	0.550	0.700	0.933	0.883
DisEn	Baseline	0.578	0.702	0.733	0.671	0.543	0.533	0.833	0.683	0.467	0.867	0.550
	EEG-ChTABNet	0.733	0.823	0.800	0.933	0.750	0.600	0.933	0.650	0.800	0.727	0.743
DistrEn	Baseline	0.578	0.648	0.867	0.543	0.475	0.400	0.900	0.767	0.467	0.933	0.533
	EEG-ChTABNet	0.667	0.827	0.867	0.800	0.720	0.600	0.900	0.783	0.550	0.787	0.703
FDispEn	Baseline	0.578	0.691	0.667	0.771	0.553	0.467	0.867	0.533	0.600	0.733	0.570
	EEG-ChTABNet	0.689	0.819	0.867	0.800	0.740	0.733	0.767	0.653	0.483	0.960	0.750
FuzEn	Baseline	0.533	0.578	0.800	0.710	0.440	0.200	0.967	0.400	0.617	0.627	0.567
	EEG-ChTABNet	0.600	0.779	0.667	0.867	0.650	0.533	0.800	0.790	0.600	0.727	0.460
LinEn	Baseline	0.600	0.739	0.733	0.700	0.608	0.467	0.933	0.467	0.617	0.760	0.550
	EEG-ChTABNet	0.689	0.781	0.867	0.705	0.637	0.600	0.900	0.733	0.617	0.927	0.850
SyDy	Baseline	0.556	0.767	0.733	0.705	0.560	0.467	0.800	0.483	0.483	0.833	0.450
	EEG-ChTABNet	0.733	0.857	0.867	0.876	0.820	0.533	0.900	0.633	0.817	0.820	0.737

**Table 4:** The CIA block means channel significant weights in SyDy and DisEn feature sets for dementia, stroke, and healthy control. Electrodes are grouped by anatomical area in the conventional 10–20 method. The values are displayed as the Mean ± SD.

Anatomical Region	Electrode	Dementia	Stroke	Control
Prefrontal	Fp1	0.763 ± 0.041	0.512 ± 0.063	0.498 ± 0.057
	Fp2	0.748 ± 0.038	0.521 ± 0.059	0.503 ± 0.061
Frontal	F3	0.791 ± 0.034	0.538 ± 0.055	0.487 ± 0.048
	Fz	0.774 ± 0.037	0.548 ± 0.061	0.494 ± 0.052
	F4	0.769 ± 0.039	0.531 ± 0.058	0.491 ± 0.050
	F7	0.612 ± 0.067	0.543 ± 0.072	0.476 ± 0.063
	F8	0.607 ± 0.071	0.537 ± 0.068	0.481 ± 0.059
Central	C3	0.543 ± 0.058	0.763 ± 0.089	0.512 ± 0.046
	Cz	0.531 ± 0.062	0.748 ± 0.094	0.508 ± 0.049
	C4	0.538 ± 0.060	0.757 ± 0.091	0.517 ± 0.044
Temporal	T3	0.624 ± 0.073	0.687 ± 0.104	0.486 ± 0.058
	T4	0.618 ± 0.069	0.671 ± 0.097	0.492 ± 0.055
	T5	0.497 ± 0.058	0.561 ± 0.088	0.598 ± 0.062
	T6	0.491 ± 0.061	0.554 ± 0.083	0.591 ± 0.064
Parietal	P3	0.463 ± 0.055	0.698 ± 0.087	0.712 ± 0.048
	Pz	0.458 ± 0.053	0.681 ± 0.091	0.697 ± 0.051
	P4	0.467 ± 0.057	0.693 ± 0.085	0.706 ± 0.049
Occipital	O1	0.412 ± 0.049	0.448 ± 0.071	0.724 ± 0.043
	O2	0.408 ± 0.051	0.441 ± 0.068	0.718 ± 0.045

**Table 5:** Ablation study results on the SyDy feature. ‘A’ is a component present and ‘x’ is a component absent.

Variant	CIA	Dual-Branch	Gated Fusion	Bottleneck Head	Acc (%)	AUC
V1: Baseline Transformer	x	x	x	x	57.8	0.748
V2: +CIA only	A	x	x	x	62.2	0.779
V3: +Dual-Branch only	x	A	x	x	64.4	0.793
V4: +Gated Fusion only	x	A	x (concat)	x	62.9	0.781
V5: +Bottleneck Head only	x	x	x	A	60.0	0.764
V6: CIA + Dual-Branch	A	A	x	x	68.9	0.821
V7: CIA + Dual + Gated	A	A	A	x	71.1	0.841
EEG-ChTABNet (Full)	A	A	A	A	73.3	0.857

**Table 6:** The mean ± SD and 95% CI for accuracy and AUC for the four best-performing feature sets for EEG-ChTABNet derived from the fold level.

Features	Metric	Fold 1	Fold 2	Fold 3	Fold 4	Fold 5	Mean ± SD	95% CI
AAPE	Acc	0.222	0.333	0.778	0.667	1.000	0.600 ± 0.320	0.600 ± 0.398
	AUC	0.574	0.352	0.778	0.759	1.000	0.693 ± 0.243	0.693 ± 0.302
AttEn	Acc	0.778	0.556	0.778	0.556	0.778	0.689 ± 0.122	0.689 ± 0.151
	AUC	0.796	0.741	0.926	0.852	0.896	0.842 ± 0.075	0.842 ± 0.093
DisEn	Acc	0.889	0.222	1.000	0.667	0.889	0.733 ± 0.310	0.733 ± 0.385
	AUC	0.852	0.426	1.000	0.926	0.911	0.823 ± 0.228	0.823 ± 0.283
DistrEn	Acc	0.778	0.333	0.778	0.778	0.667	0.667 ± 0.193	0.667 ± 0.239
	AUC	0.833	0.426	0.981	0.981	0.913	0.827 ± 0.232	0.827 ± 0.288
FDispEn	Acc	0.778	0.222	0.778	0.889	0.778	0.689 ± 0.265	0.689 ± 0.330
	AUC	0.815	0.463	0.963	0.963	0.891	0.819 ± 0.208	0.819 ± 0.258
FuzEn	Acc	0.778	0.222	0.667	0.444	0.889	0.600 ± 0.268	0.600 ± 0.332
	AUC	0.963	0.370	0.926	0.759	0.876	0.779 ± 0.241	0.779 ± 0.299
LinEn	Acc	1.000	0.222	0.667	0.778	0.778	0.689 ± 0.288	0.689 ± 0.357
	AUC	1.000	0.352	0.778	0.889	0.886	0.781 ± 0.252	0.781 ± 0.313
SyDy	Acc	0.778	0.667	1.000	0.667	0.556	0.734 ± 0.168	0.734 ± 0.209
	AUC	0.889	0.741	1.000	0.870	0.785	0.857 ± 0.100	0.857 ± 0.125

**Table 7:** The mean ± SD and 95% CI for accuracy and AUC for the four best-performing feature sets for Baseline Transformer derived from the fold level.

Features	Metric	Fold 1	Fold 2	Fold 3	Fold 4	Fold 5	Mean ± SD	95% CI
AAPE	Acc	0.333	0.222	0.667	0.667	0.333	0.444 ± 0.208	0.444 ± 0.258
	AUC	0.611	0.463	0.537	0.870	0.655	0.627 ± 0.154	0.627 ± 0.191
AttEn	Acc	0.333	0.778	0.778	0.333	1.000	0.644 ± 0.298	0.644 ± 0.370
	AUC	0.648	0.889	0.796	0.407	1.000	0.748 ± 0.230	0.748 ± 0.286
DisEn	Acc	0.222	0.222	0.889	0.667	0.889	0.578 ± 0.337	0.578 ± 0.419
	AUC	0.556	0.407	0.981	0.667	0.897	0.702 ± 0.237	0.702 ± 0.295
DistrEn	Acc	0.667	0.444	0.556	0.444	0.778	0.578 ± 0.145	0.578 ± 0.180
	AUC	0.648	0.426	0.407	0.759	1.000	0.648 ± 0.247	0.648 ± 0.306
FDispEn	Acc	0.556	0.222	0.556	0.667	0.889	0.578 ± 0.241	0.578 ± 0.299
	AUC	0.667	0.463	0.889	0.556	0.878	0.691 ± 0.190	0.691 ± 0.236
FuzEn	Acc	0.556	0.222	0.444	0.778	0.667	0.533 ± 0.214	0.533 ± 0.266
	AUC	0.667	0.185	0.593	0.722	0.725	0.578 ± 0.226	0.578 ± 0.281
LinEn	Acc	1.000	0.222	0.333	0.667	0.778	0.600 ± 0.320	0.600 ± 0.398
	AUC	1.000	0.574	0.463	0.833	0.824	0.739 ± 0.217	0.739 ± 0.269
SyDy	Acc	0.333	0.222	0.889	0.556	0.778	0.556 ± 0.283	0.556 ± 0.352
	AUC	0.556	0.481	1.000	0.852	0.944	0.767 ± 0.234	0.767 ± 0.291

**Table 8:** A comparative analysis of EEG-ChTABNet to the relevant state-of-the-art studies. Methods differ in dataset, task, and cohort size.

Study	Method	Task	Accuracy	AUC
Jeong et al. [14]	Sample Entropy, SVM	Dementia vs. Control	84.2 %	0.871
Sharma et al. [37]	EEG spectral, CNN	Stroke Detection	79.6 %	0.812
Lawhern et al. [19]	EEGNet	Motor imagery BCI	81.3 %	0.834
Craik et al. [38]	EEG Features, LSTM	Neurological	74.8 %	0.796
Song et al. [20]	EEGConformer	EEG Decoding	83.9 %	0.851
Xinchen et al. [12]	DBTSPNet Transformer	MI Classification	86.1 %	0.879
Ours: EEG-ChTABNet	Dual-branch Transformer	3-class: Dementia, Stroke, Control	73.3 %	0.857

## Data Availability

The data are not publicly available due to privacy or ethical restrictions.
